# Bifacial dye-sensitized solar cells: A strategy to enhance overall efficiency based on transparent polyaniline electrode

**DOI:** 10.1038/srep04028

**Published:** 2014-02-07

**Authors:** Jihuai Wu, Yan Li, Qunwei Tang, Gentian Yue, Jianming Lin, Miaoliang Huang, Lijian Meng

**Affiliations:** 1 Institute of Materials Physical Chemistry, Huaqiao University, 362021 Quanzhou, P. R. China; 2 Institute of Materials Science and Engineering, Ocean University of China, Qingdao 266100, P. R. China; 3 Departamento de Física, Instituto Superior de Engenharia do Porto, Instituto Politécnico do Porto, Rua Dr. António Bernardino de Almeida 431, 4200-072 Porto, Portugal, Centro de Física, Universidade do Minho, 4800-058 Guimarães, Portugal

## Abstract

Dye-sensitized solar cell (DSSC) is a promising solution to global energy and environmental problems because of its clean, low-cost, high efficiency, good durability, and easy fabrication. However, enhancing the efficiency of the DSSC still is an important issue. Here we devise a bifacial DSSC based on a transparent polyaniline (PANI) counter electrode (CE). Owing to the sunlight irradiation simultaneously from the front and the rear sides, more dye molecules are excited and more carriers are generated, which results in the enhancement of short-circuit current density and therefore overall conversion efficiency. The photoelectric properties of PANI can be improved by modifying with 4-aminothiophenol (4-ATP). The bifacial DSSC with 4-ATP/PANI CE achieves a light-to-electric energy conversion efficiency of 8.35%, which is increased by ~24.6% compared to the DSSC irradiated from the front only. This new concept along with promising results provides a new approach for enhancing the photovoltaic performances of solar cells.

Solar cells have been at the heart of current low-carbon economy and have led to growing interest in the field of environmental and energy applications because of the depletion and pollution of fossil fuels^1^,^2^,^3^. Among various solar cells, dye-sensitized solar cell (DSSC) is a promising candidate owing to its clean, low cost, easy preparation, good durability, and high conversion efficiency^4^,^5^,^6^,^7^,^8^. A typical DSSC consists of three components: a dye-sensitized nanocrystalline TiO_2_ photoanode, iodide/triiodide redox electrolyte, and a counter electrode (CE). The CE is a conducting layer with electrocatalytic function that serves to catalyze the redox couple regeneration reaction and collect electrons from external circuit. Hereinto, platinum (Pt) CE is preferred because of its high electrocatalytic activity to iodide/triiodide redox reaction and good conductivity. However, Pt is a noble metal; it needs to develop CE alternatives with low-cost, high conductivity and good electrocatalytic activity. Many functional materials^9^,^10^,^11^ have been replaced Pt as CEs, among them, polyaniline (PANI) is a promising candidate due to its excellent properties, such as good electrocatalytic activity, high conductivity, low cost, easy preparation and environmental stability.

On the other hand, in order to increase the incident light harvest and thus enhance the conversion efficiency of DSSC, a scattering layer on TiO_2_ anode^12^,^13^,^14^ and a mirror face^15^,^16^ on the CE have been devised; however, the processing procedure and actual results are still not so satisfactory. Based on the dilemma, an inspiration is aroused from the bifacial silicon solar cells^17^, where a front efficiency of 17.0% (irradiation from the front, 100 mW·cm^−2^) and a rear efficiency of 7.1% (irradiation from the rear, 42 mW·cm^−2^), giving an enhanced total efficiency of 24.0%. Recently, a bifacial DSSC consisted of TiO_2_/SiO_2_ anode and Pt CE was reported by Gratzel *et al*, a power conversion efficiency of ~6% was obtained for the DSSC irradiated from the front or the rear side^18^. The more recent research by Zhao *et al* reported a bifacial DSSC with a TiO_2_ anode and a transparent PANI CE^19^, yielding a front-illuminated power conversion efficiency of 6.54% and a rear-illuminated efficiency of 4.26%.

In a DSSC, incident sunlight must penetrate through conductive glass substrate and excite the dye molecules on TiO_2_ film. Although most part of incident light is absorbed by the dye molecules, small part of incident light directly pass through and is not utilized in DSSC. In this study, we design a bifacial DSSC by combining transparent PANI counter electrode with traditional TiO_2_ photoanode (shown in Fig. 1). The incident light perpendicularly irradiates on the front side of the DSSC, and the transmitted light through the DSSC and round the edges of the device is reused by reflecting it onto the rear side of the DSSC. Owing to transparent counter electrode, the rear light can penetrate and be absorbed by the dye molecules in the DSSC, which will enhance the short-circuit current density and thus overall conversion efficiency. It is expected^17^,^18^,^19^ that the bifacial DSSC can increase overall conversion efficiency of 20 ~ 40%.

## Results

### The structure of bifacial DSSC

**Figure 1:**
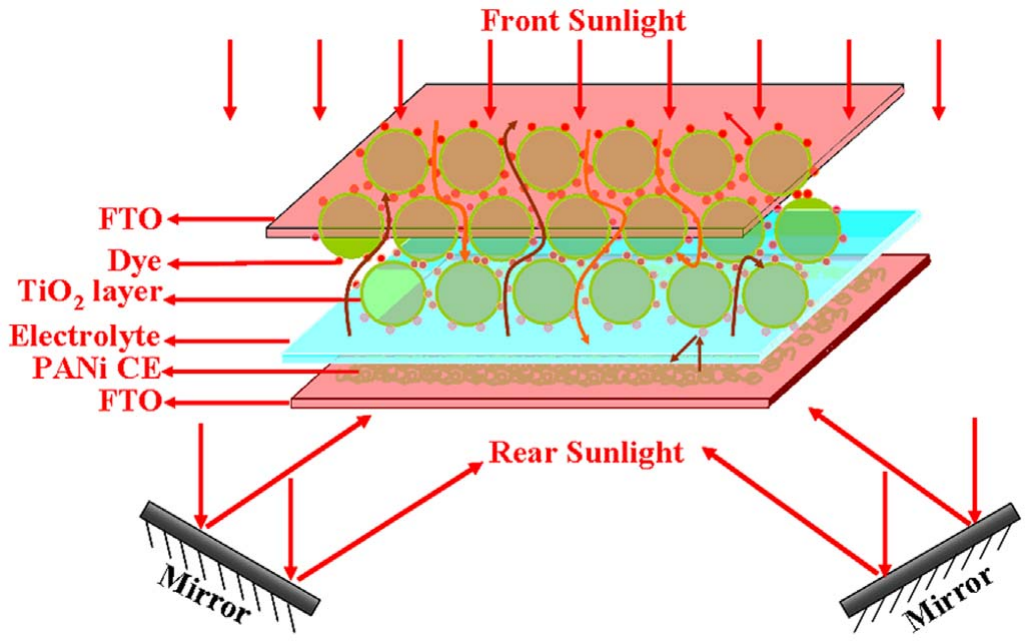
Schematic illustration of the light-splitting design and simulated sunlight tunnels under the irradiation from both the front and the rear. The incident light intensities from the front and the rear are 100 and 68 mW·cm^−2^, respectively.

The schematic structure of the bifacial DSSC is shown in Fig. 1. FTO conductive glass substrate is coated with TiO_2_ nanoparticle film. The TiO_2_ film is sensitized with N719 dye and used as a photoactive electrode, whereas PANI film is transparent and serves as an electrocatalytic CE. iodide/triiodide redox electrolyte is filled between the two electrodes. To realize the bifacial irradiation, the incident sunlight is divided into two beams via a light-splitting design. The incident light irradiation intensities from the front and the rear are 100 and 68 mW·cm^−2^, respectively. The synergetic effect of the front-side and the rear-side irradiations results in the sufficient utilization of sunlight and the enhancement of power conversion efficiency.

### The morphology of PANI

**Figure 2:**
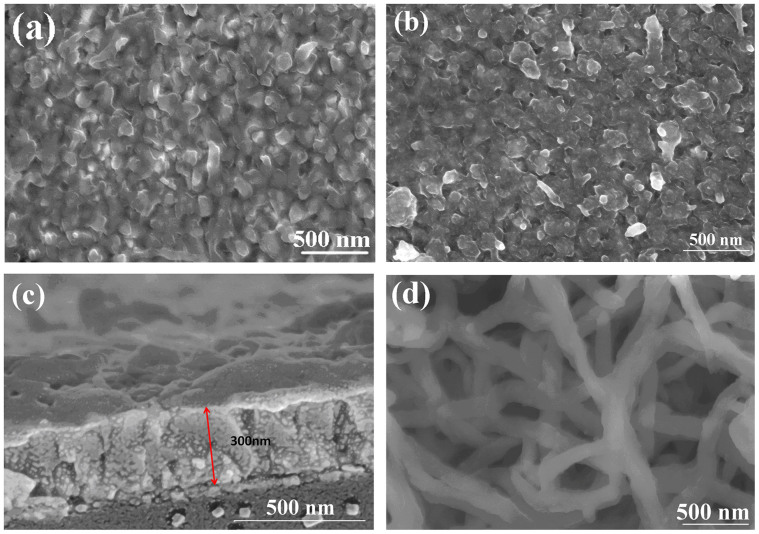
FESEM images of (a) PANI (CD, top view), (b) 4-ATP/PANI (CD, top view), (c) 4-ATP/PANI (CD, cross-sectional view), and (d) PANI (ECD, top view).

PANI film was prepared by chemical deposition (CD) method^19^ on a FTO substrate. To diminish the interfacial resistance and accelerate the charge transport between PANI and FTO glass substrate, 4-aminothiophenol (4-ATP) was used^20^ to form 4-ATP/PANI film. Fig. S1 shows the XPS spectra of bared FTO and 4-ATP modified FTO substrates, the latter has stronger peaks at ~285 and ~400 eV for C = C and –NH_2_ groups^21^,^22^, implying that the 4-ATP is bonded on the FTO. On the other hand, according to the references^23^,^24^, –SH can be well assembled on the FTO surface. So it is believed that 4-ATP as a bridging agent improves the combination of FTO layer via –SH end and guide the growth of PANI chains via −NH_2_ end to form 4-ATP/PANI film^25^. Figs. 2a and 2b show the typical top-view FESEM images of FTO supported PANI (CD) and 4-ATP/PANI (CD) films. Compared with PANI (CD) film, the 4-ATP/PANI (CD) film shows more porous and rough surface, which can be validated by the cross-section view shown in Fig. 2c. The microporous structure fortifies the contact with FTO, favors the permeation of iodide/triiodide electrolyte, and thus accelerates charge transportation. As a comparison, PANI was prepared on FTO substrate by electrochemical deposition (ECD) method, its morphology is observed in Fig. 2d. The nanofiber shape and porous structure for this PANI (ECD) is believed to be a better electrocatalytic performance to iodide/triiodide electrolyte. However, the separation of the PANI from FTO substrate (shown in Fig. S2) offers a high interfacial resistance, which is an unfavorable factor for charge transportation.

### The optical properties of CEs

**Figure 3:**
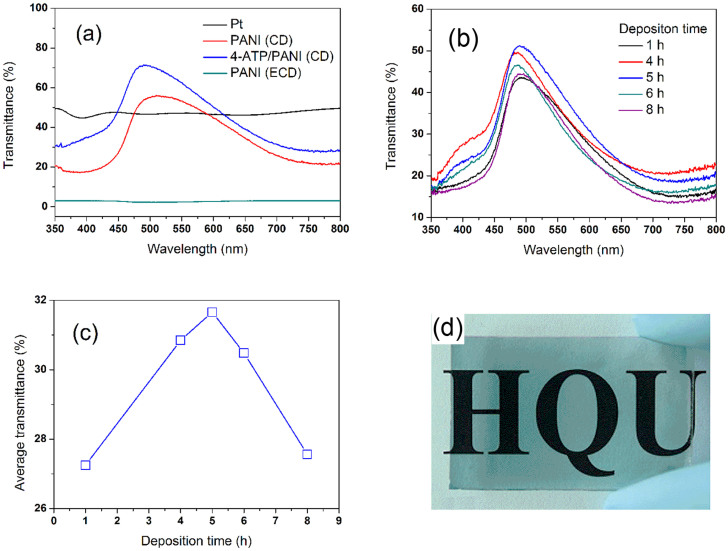
(a) UV-vis transmittance spectra of four CEs, (b) UV-vis transmittance spectra of PANI (CD) prepared in different deposition time, (c) the relationship between transmittance and deposition time, (d) photograph of 4-ATP/PANI CE.

The transmittance of CE is an important factor affecting incident light harvest from the rear and the conversion efficiency of the bifacial DSSC. Fig. 3a compares the transmittances of the four kinds of CEs. The PANI(ECD) CE has the smallest transmittance, which is due to that the fiber-shape PANI blocks the transmission of light. PANI (CD) CE shows the better transmittance in wavelength range 350–800 nm, especially in the deposition time of 5 h (Figs. 3b and 3c). This is due to that in the shorter deposition time, an even PANI layer can not be formed, only some PANI particles deposit on the FTO, which results in the lower transmission. On the other hand, the longer deposition time produces the thicker PANI film on the FTO, which decreases the transmission of light. According to experimental results, under our conditions, the deposition time of 5 h is optimal (Fig. 3c). Interesting, the deposition time of 5 h gives the PANI (CD) CE the best electrocatalytic activity (will be discussed follow), so the deposition time of 5 h is adopted as an optimal preparation condition for PANI (CD) CE. The transmittance of PANI (CD) electrode is further improved by modifying with 4-ATP (Figs. 3a and 3d). This is due to that 4-ATP as a bridging agent improves the combination of FTO layer via –SH end and guide the growth of PANI chains via −NH_2_ end to form 4-ATP/PANI film. Although Pt CE also has better average transmittance in wavelength range 350–800 nm, the 4-ATP/PANI (CD) CE shows the higher transmittance in dye 719 sensitized range 450–620 nm (Figs. 3a and 3d). Therefore 4-ATP/PANI is a better candidate than Pt as CE for the bifacial DSSC.

### The electrical properties of CEs

**Figure 4:**
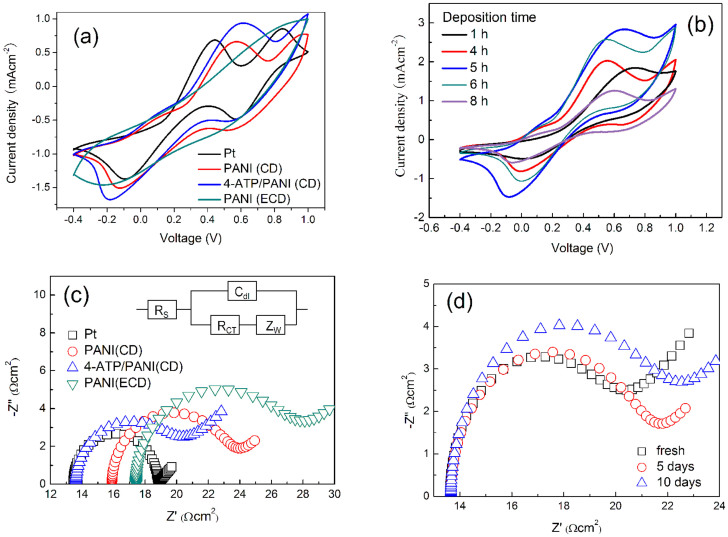
(a) Cyclic voltammograms for Pt, PANI (CD), 4-ATP/PANI (CD), and PANI (ECD) electrodes recorded in a supporting electrolyte consisting of 10 mM LiI, 1 mM I_2_ acetonitrile solution containing 0.1 M LiClO_4_ at a scan rate of 50 mV·s^−1^. (b) Cyclic voltammograms for PANI (CD) CEs with different deposition times in the same electrolyte as (a). (c) Nyquist plots of Pt, PANI (CD), 4-ATP/PANI (CD), and PANI (ECD) electrodes obtained at open circuit voltage under irradiation of 100 mW·cm^−2^. The inset is the equivalent circuit. (d) Nyquist plots of EIS for the symmetrical cells with 4-ATP/PANI (CD) electrode subjected to aging at room temperature.

Cyclic voltammetry (CV) was used to investigate electrocatalytic activity of CEs toward to iodide/triiodide (I^−^/_3_^−^) redox couple. The CE in DSSC serves as an electrocatalyst for reducing I_3_^−^ to I^−^ (I_3_^−^ + 2e → 3I^−^), therefore, the cathodic reduction peak current density in CV curves can be used to evaluate the electrocatalytic activity of the CEs^9^,^19^. From Fig. 4a, the cathodic reduction peak current densities for four samples are in the order: 4-ATP/PANI (CD) > PANI (CD) > PANI (ECD) ≈ Pt. The results indicate that the electrocatalytic activity of PANI (CD) is better than that of Pt and PANI (ECD). The deposition time of 5 h gives PANI (CD) electrode the best electrocatalytic activity (Fig. 4b). This is because the shorted deposition time can not produce enough catalytic active sites, and longer deposition time generates an increased film thickness and resistance for charge transfer. The electrocatalytic activity of PANI (CD) is further enhanced by modification of 4-ATP on PANI (Fig. 4a). It is notable that the 4-ATP/PANI (CD) and PANI (CD), especially for PANI (ECD), electrodes have higher overpotential for the reduction of I_3_^−^ then Pt electrode does from the reduction peak position in Fig. 4a. However, owing to the bridge effect by 4-ATP, 4-ATP/PANI (CD) electrode has the largest peak current density, which compensates its disadvantage in overpotential. Therefore, the 4-ATP/PANI (CD) is adopted as an efficient CE in the bifacial DSSC.

The EIS measurement was performed in a symmetrical dummy cell fabricated with two identical CEs and using the same electrolyte for the bifacial DSSC. Fig. 4c shows the Nyquist plots obtained from the dummy cell, relative series resistance (R_S_) and charge transfer resistance (R_CT_) on counter electrode/electrolyte interface^26^,^27^ are listed in Table 1. It can see that PANI (ECD) electrode has a highest R_S_ (17.42 Ω·cm^2^) and R_CT_ (5.07 Ω·cm^2^), indicating an unsuitable CE in DSSC. The PANI (CD) electrode has moderated R_S_ (15.89 Ω) and R_CT_ (3.65 Ω·cm^2^); when PANI (CD) is modified with 4-ATP, the *R_S_* and *R_CT_* are decreased to 13.61 and 3.20 Ω·cm^2^, respectively, almost the same as the Pt electrode (13.44 and 2.69 Ω·cm^2^). Lower R_CT_ for 4-ATP/PANI is due to the higher electrocatalytic activity to redox electrolyte (which is verified by Fig. 4a) and the better interfacial ohmic contact between 4-ATP/PANI and FTO substrate compared to PANI (CD) and PANI (ECD) electrodes. Since the 4-ATP can be used as an effective bridge across FTO substrate and PANI by −NH_2_ group on 4-ATP with PANI and −SH group on 4-ATP with FTO substrate. Therefore, the R_CT_ on counter electrode/electrolyte interface is decreased. Fig. 4d shows the impedance spectra for the fresh and aged dummy cells from 4-ATP/PANI (CD) electrode. There is a reasonable stability for R_s_, which indicates that 4-ATP/PANI (CD) electrode has a good stability in redox electrolyte solution. By contrast, the R_ct_ increases from 3.20 to 4.5 Ω·cm^2^ after aging for 10 days. This indicates a decrease in transferring charges.

### The photovolatic performance of bifacial DSSCs

**Figure 5:**
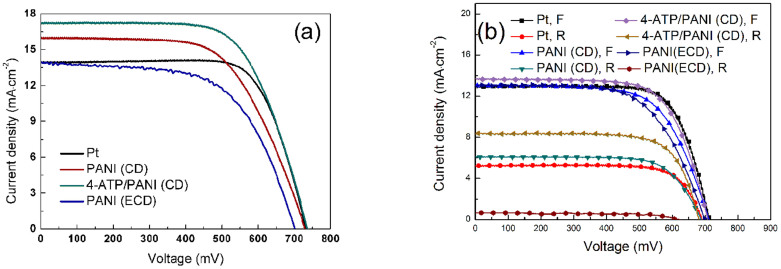
(a) *J-V* curves of the bifacial DSSCs using Pt, PANI (CD), 4-ATP/PANI (CD), and PANI (ECD) as CEs under simulated sunlight irradiation from the both of front (100 mW·cm^−2^) and rear (68 mW·cm^−2^), (b) *J-V* curves of the bifacial DSSCs using four kinds of CEs under simulated sunlight irradiation (100 mW·cm^−2^) from only the front (F) or the rear (R).

Fig. 5a shows the *J-V* curves of the bifacial DSSCs using Pt, PANI (CD), 4-ATP/PANI (CD), and PANI (ECD) as CEs under simulated sunlight irradiation from the both of front and rear sides. Fig. 5b shows the *J-V* curves of the bifacial DSSCs irradiated only from the front or rear side. The results are summarized in Table 1. When irradiated from front side, the DSSC using Pt CE exhibits an efficiency of 6.80%, which is the average level of literatures^28^,^29^,^30^,^31^. However, a lower efficiency of 2.73% is obtained by irradiation from the rear side because of a high reflectivity of Pt CE. The reduced *J*_SC_ is due to the decrease in incident light intensity via the reflection of Pt CE, thus diminishes the photogenerated charge carriers and light-to-electric conversion efficiency^19^. The enhanced J_SC_ from both-side illumination benefits from the increased accumulative photogenerated electron density. Interestingly, the bifacial DSSC with Pt CE irradiated from both front and rear sides obtains an efficiency of 7.44%. Although Pt CE possesses high reflection, the transmitted light from the rear can produce photogenerated charge carriers and photocurrent, resulting in higher efficiency for both-side irradiation than one-side irradiation. The bifacial DSSC with PANI (ECD) CE irradiated from the both sides obtains a low efficiency of 5.90%. If the cell is irradiated only from the front or the rear, the efficiency are 5.79% and 0.28%, respectively. Low efficiency for the DSSC with PANI (ECD) CE is due to its CE low electrocatalytic activity (Fig. 4a) and high resistances (R_CT_ and R_S_, Fig. 4c), especially, the lowest efficiency (0.28%) for the DSSC with PANI (ECD) CE irradiated from the rear comes from the lowest transmittance (2 ~ 3%, Fig. 3a). The bifacial DSSC with PANI (CD) CE irradiated from the both sides obtains an efficiency of 7.05%. When the cell is irradiated only from the front or the rear, the efficiency are 6.11% and 3.01%, respectively.

Compared with the former, the efficiency of the DSSC with PANI (CD) CE irradiated from the rear is remarkably enhanced from 0.28% to 3.01%, the mainly reason is the higher transmittance of PANI (CD) CE than that of PANI (ECD) CE (Fig. 3a).

Using 4-ATP/PANI (CD) as CE, the bifacial DSSC obtains highest both-side efficiency (8.35%) and rear-side efficiency (4.15%). The both-side efficiency is increased by 18.44% and rear-side efficiency is increased by 37.87%, compared with the DSSC with PANI (CD) CE. Among four kinds of DSSCs, the bifacial DSSC with 4-ATP/PANI (CD) CE shows the highest light-to-electric energy conversion efficiency, which is attributed to fine microporous structure, high transmittance, good electrocatalytic activity to I_3_^−^ reduction, low charge transfer resistance on electrolyte/electrode interface for 4-ATP/PANI (CD) CE. The monochromatic incident photon-to-electron conversion efficiency (IPCE) curves for 4-ATP/PANI (CD)-based and Pt-based DSSCs were measured and shown in Figure S6. According to this Figure, the 4-ATP/PANI-based DSSC has better photovoltaic performance than the Pt-based DSSC does. The both-side efficiency (8.35%) of DSSC with 4-ATP/PANI CE is enhanced by 24.6% compared to the front-side efficiency. The efficiency value (8.35%) has been the highest record for the DSSC using PANI as CE, so far. It is also believed that the value can be further improved by designing a better light-splitting device with vertical incidence. The new concept and therefore significant enhancement in efficiency can be applied other DSSCs with transparent CEs such as PANI, PEDOT:PSS, and graphene.

## Discussion

In summary, incident light loss and therefore incomplete excitation of dyes drive us to design a bifacial DSSC that can be simultaneously irradiated from both the front and rear sides. In this work, a transparent PANI film was prepared on FTO substrate by a simple and low-cost chemical deposition method. To accelerate the charge transfer from FTO to PANI, 4-ATP was used as a bridging agent via a covalent bonding between FTO and −SH end on 4-ATP and an in-situ growth of PANI chain on −NH_2_ end on 4-ATP, showing a much better electrochemical behavior to iodide/triiodide. According to the characterization by FESEM, UV-vis, CV and EIS, the 4-ATP/PANI (CD) electrode shows fine microporous structure, high transmittance, good electrocatalytic activity to triiodide reduction, low charge transfer resistance on electrolyte/electrode interface. The bifacial DSSC based on 4-ATP/PANI CE achieved an overall light-to-electric energy conversion efficiency of 8.35%, which is the highest record in PANI CE-based DSSCs. This new concept and promising results provide a new route for enhancing the photovoltaic performance of solar cells. The design concept of bifacial DSSC can be used in other photovoltaic systems with transparent CEs.

## Methods

### Modification of FTO glass by 4-ATP

A self-assembly technique^20^ was used to modify FTO conductive glass (Fluorine-doped tin oxide, sheet resistance 8 Ω/≤, purchased from Hartford Glass Co. USA) to form a uniform 4-ATP (purity 97%, purchased from Sinopharm Chemical Reagent Co., Ltd, China) monolayer. The details were described as follows: The FTO glass was cut into 2.5 × 1.5 cm^2^ and was cleaned successively by an ultrasonic bath in deionized water, isopropanol, acetone and ethanol for 30 min, respectively. After being dried in an oven, the substrates were immersed in 4-ATP/ethanol solution ([4-ATP] = 0.03 M) for more than 1 h. Finally, the FTO substrates were rinsed with deionized water and used for PANI deposition.

### The preparation of PANI CE by chemical deposition

Transparent PANI CEs were prepared as following^19^: 100 ml of 25 g·L^−1^ ammonium peroxydisulfate (APS) aqueous solution was slowly dropped into the mixture of aniline (99.5%, purchased from Sinopharm Chemical Reagent Co., Ltd, China) solution consisting of aniline monomer of 1 mL and H_2_SO_4_ (95 ~ 98%) of 6 mL. This procedure accelerates the decomposition of APS and polymerization of aniline to PANI. After vigorous agitation, the FTO glass substrate was immersed in the reagent without agitating over 5 h at ambient temperature. After the polymerization, the attached PANI molecules on the nonconductive side were removed and the whole PANI film was rinsed by enough deionized water and ethanol. Finally, the PANI electrode was dried in a vacuum oven at 60°C for 24 h. As a comparison, the Pt film was also electrochemically deposited on a bared FTO glass substrate according to reference^28^.

### The preparation of PANI CE by electrochemical deposition

For comparison, PANI was prepared by an electrochemical deposition method. The PANI CE was prepared in a three-electrode system on an electrochemical workstation (CHI660C, CH Instruments, Shanghai) according to the reference^32^. A FTO substrate was used as the working electrode, a platinum wire as the counter-electrode and a saturated Ag/AgCl as reference electrode. Anodic deposition was controlled in a solution containing 1.0 M HClO_4_ and 0.2 M aniline monomer. The PANI CE was prepared by controlling the number of sweep segments and with initial E = −0.2 V, high E = 0.9 V, low E = −0.2 V, final E = 0.7 V, scan rate = 50 mV·s^−1^, segment = 141. The resultant PANI CE was washed with distilled water and ethanol followed by drying in a vacuum oven at 60°C for 4 h.

### Preparation of TiO_2_ film and fabrication of the bifacial DSSCs

The cleaned FTO glass substrate was immerged in 0.15 M TiCl_4_ isopropanol solution for 12 h, followed by sintering in air at 450°C for 30 min^33^. A TiO_2_ colloid was prepared according to previously reported method^8^,^34^,^35^. A thin TiO_2_ blocking layer was firstly deposited onto the well-cleaned FTO substrate. Then, a TiO_2_ film with a 10-μm-thick and particles size of 10–20 nm layer was prepared by coating the TiO_2_ colloid using a doctor blade technique, followed by sintering in air at 450°C for 30 min. Subsequently, the TiO_2_ films were soaked in a 0.3 mM N719 [cis-di(thiocyanato)-N,N′-bis(2,2′-bipyridyl-4-carboxylic acid-4-tetrabutylammonium carboxylate, purchased from Solaronix, SA, Switzerland] ethanol solution for 24 h to uptake N719 dye for the fabrication of dye-sensitized TiO_2_ photoanode. The DSSC was assembled by sandwiching the electrolyte (0.05 M I_2_, 0.1 M LiI, 0.6 M tetrabutylammonium iodide and 0.5 M TBP in acetonitrile) between the TiO_2_ photoanode and PANI CE^8^,^34^,^35^.

### Characterizations and measurements

UV-vis transmittance spectra of samples were recorded on UV-2550 spectrometer (Shimadzu, Japan). SEM image of the sample were recorded with an S-4800 instrument equipped with an INCA 7201 EDS at an acceleration voltage of 5 kV. Atomic force microscopy (AFM) was conducted on a Nanoscope Multimode III a scanning probe microscopy system in tapping mode. The cyclic voltammetry (CV) of PANI electrode was recorded on a traditional three-electrode electrochemical workstation (CHI660C, CH55 Instruments, Shanghai) at a scan rate of 50 mV·s^−1^ using the prepared electrode as working electrode, a Pt-foiled as CE and a saturated Ag/AgCl as reference electrode. The electrolyte was a mixture of an acetonitrile solution of 100 mM LiClO_4_, 10 mM LiI, and 1 mM I_2_. The electrochemical impedance spectroscopy (EIS) measurement was carried out on the CHI660 C electrochemical workstation with the method reported in the literature^11^. As for the impedance study on the CE/electrolyte interface in the symmetric dummy cells, an equivalent circuit model was used to fit the data with Zview software. The charge transfer resistance (*R*_ct_) was calculated as half the value obtained from the fitting (the cells used were symmetric) and then multiplied by the geometric area of the electrode. The measurements were performed simulating open circuit conditions (0 V applied across the dummy cell) at frequencies ranging from 0.1 Hz to 100 kHz and the ac amplitude was set at 20 mV. The photovoltaic tests of DSSCs were carried out by measuring the *J–V* characteristic curves under simulated AM 1.5 G solar illumination at 100 mW·cm^−2^ from a xenon arc lamp (CHFXM500, Trusttech Co., Ltd, China) in ambient atmosphere and recorded with CHI660C. Monochromatic incident photo-to-current conversion efficiency (IPCE) curves of devices were measured on an IPCE measurement systems (MS260). The light source in this case was a solar simulator (PEC-L11, AM1.5G, Peccell Technologies, Inc.); Light was focused through a monochromator onto the photovoltaic cell.

## Author Contributions

J.W. and Q.T. designed the device and experiments, Y.L., G.Y., J.L., M.H. and L.M. carried out all experiments. Q.T. wrote the first draft, J.W. wrote the second draft, and all authors discussed the results and contributed to revisions.

## Supplementary Material

Supplementary Information Supporting Imformation

## Figures and Tables

**Table 1:** EIS and Photoelectric parameters of the bifacial DSSCs

CE and irradiation direction	*R*_s_ (Ω·cm^2^)	*R*_ct_ (Ω·cm^2^)	*V*_OC_ (mV)	*J*_SC_ (mA·cm^−2^)	*FF*	*E*_ff_ (%)
Pt, front & rear	13.44	2.69	732	14.01	0.728	7.46
Pt, front			714	12.94	0.736	6.80
Pt, rear			690	5.23	0.758	2.73
PANI(CD), front & rear	15.89	3.65	730	15.98	0.604	7.05
PANI(CD), front			712	13.08	0.655	6.11
PANI(CD), rear			689	6.11	0.715	3.01
4-ATP/PANI(CD), front & rear	13.61	3.20	730	17.51	0.653	8.35
4-ATP/PANI(CD), front			709	13.71	0.689	6.70
4-ATP/PANI(CD), rear			682	8.34	0.729	4.15
PANI(ECD), front & rear	17.42	5.07	701	13.97	0.600	5.88
PANI(ECD), front			700	12.90	0.642	5.79
PANI(ECD), rear			622	0.66	0.684	0.28
