# Supplementary material for: Bifacial dye-sensitized solar cells: A strategy to enhance overall efficiency based on transparent polyaniline electrode
Source: Sci Rep. 2014 Feb 7;4:4028. doi: 10.1038/srep04028 (PMC3916787; doi:10.1038/srep04028)
Supplement: Supplementary Information — Supporting Imformation [file srep04028-s1.pdf]

Supporting Information

for *Scientific Report.*, DOI:10.1038/srep (2014).

Bifacial dye-sensitized solar cells: A strategy to enhance overall efficiency based on transparent polyaniline electrode

Jihuai Wu[1]*, Yan Li[1], Qunwei Tang[2]*, Gentian Yue[1], Jianming Lin[1], Miaoliang Huang[1] & Lijian Meng[3]

[1] Institute of Materials Physical Chemistry, Huaqiao University, 362021 Quanzhou, P. R. China; [2] Institute of Materials Science and Engineering, Ocean University of China, Qingdao 266100, P. R. China; [3] Departamento de Física, Instituto Superior de Engenharia do Porto, Instituto Politécnico do Porto, Rua Dr. António Bernardino de Almeida 431, 4200-072 Porto, Portugal, Centro de Física, Universidade do Minho, 4800-058 Guimarães, Portugal.

Figures S1-S6

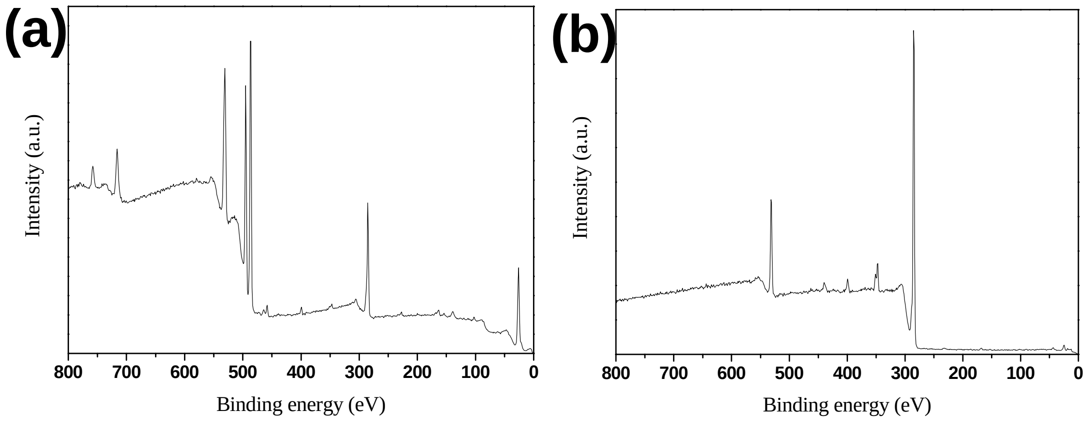

Figure S1 │ XPS spectra for (a) bared FTO and (b) 4-ATP modified FTO substrates.

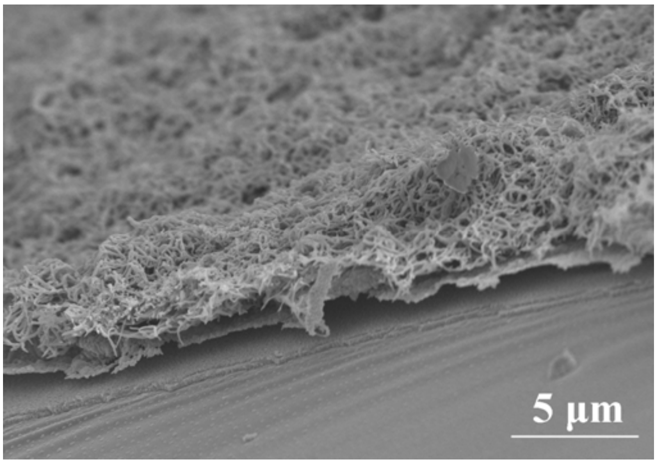

Figure S2 │ Cross-sectional SEM image of PANI (ECD) on FTO glass substrate.

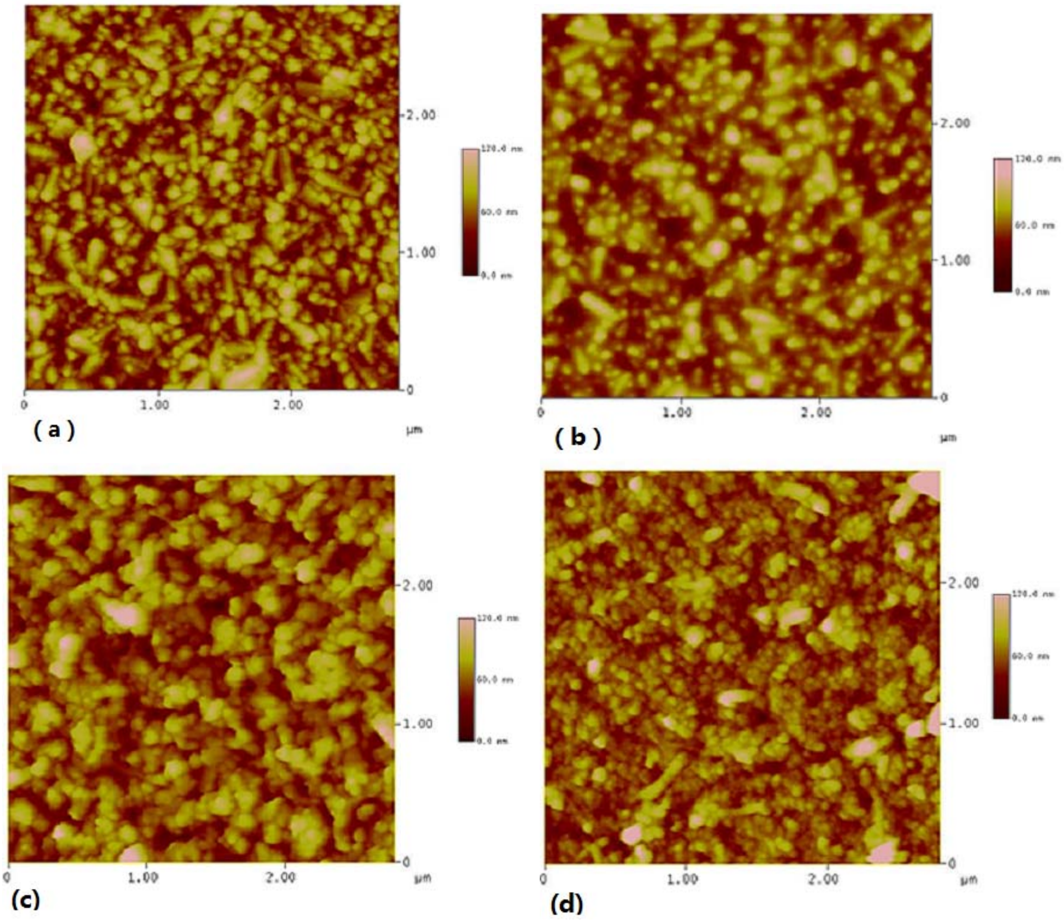

Figure S3 │ AFM images of (a) bare FTO substrate, (b) 4-ATP/FTO, (c) PANI (CD) on FTO, (d) 4-ATP/PANI (CD) on FTO.

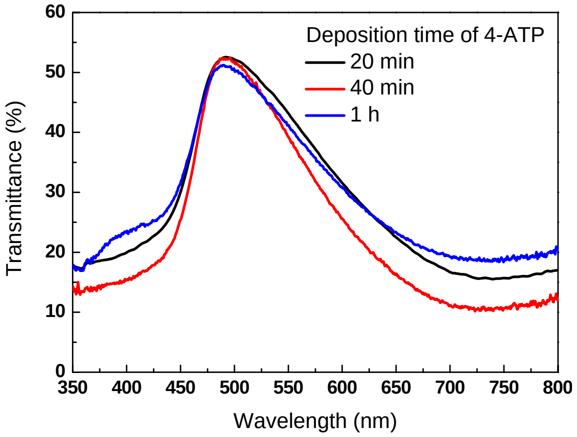

Figure S4 │ UV-vis transmittance spectra of FTO supported 4-ATP/PANI (CD) electrode at various deposition time of 4-ATP. The deposition time of PANI is 5 h.

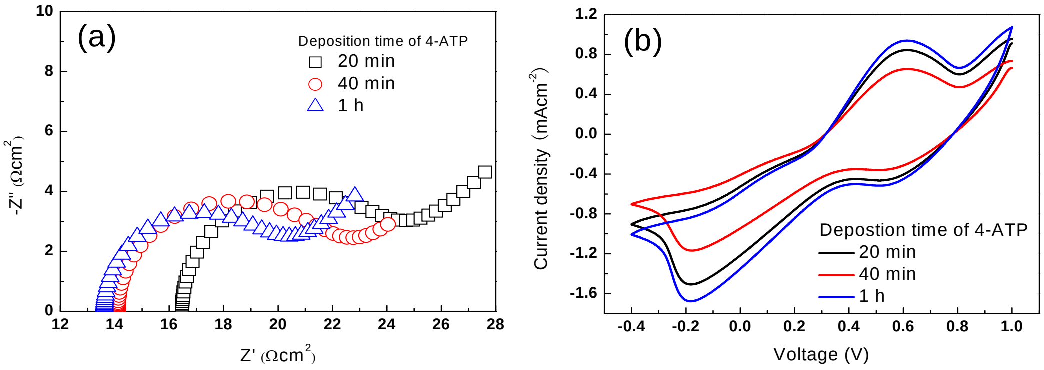

Figure S5 │ (a) Nyquist plots and (b) cyclic voltammograms of 4-ATP/PANI (CD) electrodes at various deposition times of 4-ATP. The deposition time of PANI is 5 h. The Nyquist plots were obtained at open circuit voltage under irradiation of 100 mW·cm^{-2}.

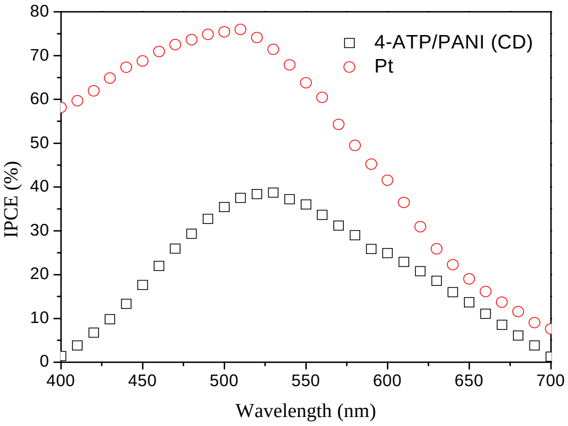

Figure S6 │ IPCE curves of DSSCs from 4-ATP/PANI (CD) and Pt counter electrodes. The deposition time of PANI is 5 h.
